# The role of the dystrophin glycoprotein complex in muscle cell mechanotransduction

**DOI:** 10.1038/s42003-022-03980-y

**Published:** 2022-09-27

**Authors:** Darren Graham Samuel Wilson, Andrew Tinker, Thomas Iskratsch

**Affiliations:** 1 School of Engineering and Materials Science, Queen Mary University of London, London, UK; 2 William Harvey Research Institute, Queen Mary University of London, London, UK

**Keywords:** Mechanotransduction, Mechanisms of disease

## Abstract

Dystrophin is the central protein of the dystrophin-glycoprotein complex (DGC) in skeletal and heart muscle cells. Dystrophin connects the actin cytoskeleton to the extracellular matrix (ECM). Severing the link between the ECM and the intracellular cytoskeleton has a devastating impact on the homeostasis of skeletal muscle cells, leading to a range of muscular dystrophies. In addition, the loss of a functional DGC leads to progressive dilated cardiomyopathy and premature death. Dystrophin functions as a molecular spring and the DGC plays a critical role in maintaining the integrity of the sarcolemma. Additionally, evidence is accumulating, linking the DGC to mechanosignalling, albeit this role is still less understood. This review article aims at providing an up-to-date perspective on the DGC and its role in mechanotransduction. We first discuss the intricate relationship between muscle cell mechanics and function, before examining the recent research for a role of the dystrophin glycoprotein complex in mechanotransduction and maintaining the biomechanical integrity of muscle cells. Finally, we review the current literature to map out how DGC signalling intersects with mechanical signalling pathways to highlight potential future points of intervention, especially with a focus on cardiomyopathies.

## Abstract (web summary)

A review of the function of the Dystrophic Glycoprotein Complex (DGC) in mechanosignaling provides an overview of the various components of DGC and potential mechanopathogenic mechanisms, particularly as they relate to muscular dystrophy.

## Introduction

Cells are in continual communication with their microenvironment and the bidirectional dialogue between the two is crucial for the interpretation and integration of biomechanical information. Biomechanics governs key downstream events (e.g., cytoskeletal rearrangement), directing the overall cellular phenotype in space and time. Central to this process in cardiomyocytes are costameric regions, the region where the sarcolemma connects to the sarcomere, comprised of integrin-talin-vinculin and the dystrophin-glycoprotein complexes (DGC). These discrete focal adhesions (FA) link to the intracellular cytoskeleton propagating a cascade of biomechanical and biochemical cellular changes governing differentiation, proliferation, organogenesis, migration, disease progression, amongst others. The conversion of biomechanical forces into a biochemical and/or (epi)genetic change is termed mechanotransduction^1^.

Integrin transmembrane receptors^2^ have been long recognised as anchoring cells to the ECM as well as mediating both inside-out and outside-in signalling. In parallel to the integrins, the DGC connects the ECM to the cellular cytoskeleton thus establishing the critical connection between the exterior and the interior of the cell^3^. Full-length dystrophin (Dp427) is expressed predominantly in cardiac and skeletal muscle, however, it has been observed in central nervous system tissues, including the retina and purkinje tissue^4^. Mutations in both integrins and the DGC have revealed themselves to be causes for muscular dystrophies and progressive dilated cardiomyopathy (DCM) (Table 1)^5,6^. Specifically, mutations in *DMD* which encodes the central protein of the DGC, dystrophin, causes Duchenne muscular dystrophy (DMD)^7^. The DGC is comprised of several subcomplexes including α-, and β-dystroglycan (α/β-DG), sarcoglycan-sarcospan, syntrophin, as well as dystrophin^8^.

**Table 1:** Overview of mutations in DGC components and integrins that cause distinct forms of muscular dystrophy.

Disease	Gene	Location	Mutation	Phenotype	Ref
Dystrophinopathy	**Deletions**				
	*DMD*	Xp21.1	ΔEx 1 (muscle promoter region)	XLCM with fibrosis; Fatal DCM	^238,239^
			ΔEx 48–50	DCM and aberrant Ca^2+^ handling	^240^
			ΔEx 4	DCM with severe fibrosis	^241^
			ΔEx 48–54	Left ventricular dysfunction with abnormal ECG. Pre-mature death	^242^
	**Duplications**				
			Ex 2 dup.	Decreased left ventricular function, hypokinesia, and DCM	^243^
			Ex 8–11 dup.	Cardiomyopathy present	^244^
	**Point Mutations**				
			c.1043 A>G (p.T279A)	Mutation in hinge 1 region of dystrophin. XLCM	^245^
			c.4996 C>T (p.Arg1,666X)	Premature stop codon. Arrhythmia and aberrant Ca^2+^ handling. Increased ROS production	^246^
			c.10801 C>T (p.Gln3601X)	Premature stop codon. Exon 76 absent. Cardiomyopathy.	^247^
Dystroglycanopathy	**Deletions**				
	*LARGE*	22q12.3	ΔEx9-10	WWS present, hypotonia and severe neurological pathology. Premature death at age 6 months	^248^
	**Point Mutations**				
	*FKRP*	19q13.3	c.296 A>G (p.Y309C)	Congenital muscular dystrophy with severe hypotonia. Cardiac phenotype not reported by Brockington.	^77^
			c.826 C>A	Mutations in *FKRP* caused LGMD2I with cardiomyopathy reported. Abnormal ECG and respiratory distress.	^249^
	*FCMD*	19q31	c.859 T>G (p.C250G)	Range of severity. Can be fatal by 1yr as in WWS or relatively mild. Cardiac involvement has been reported.	^250^
	*DAG1*	3p21	c.575 C>T (p.T192M)	LGMD with neurocognitive difficulties. No cardiac pathology was found.	^251^
	*POMT1*	9q34.1	c.430 A>G (p.N144D)	DCM onset at 12yrs with ejection fraction of 36%.	^252^
Sarcoglycanopathy	**Insertions**				
	*SGCB*	4q12	(Ex 3) 383^384ins376–383	LGMD with severe DCM	^253^
	**Point Mutations**				
	*SGCA*	17q21	c.218 C>T (p. P73L)	LGMD2D	^92^
Integrins	**Duplications**				
	*ITGA7*	12q13.2	c.1088dupG (p. H363Sfs*15)	Congenital muscular dystrophy with limb atrophy. Cardiac function was reportedly normal.	^254^
	**Point Mutations**				
			c.1506-2A>G	Congenital muscular dystrophy with severe neurocognitive difficulties	^145^

*DCM* dilated cardiomyopathy, *LGMD* Limb-Girdle muscular dystrophy, *WWS* Walker-Warburg syndrome, *XLCM* X-Linked cardiomyopathy.

Dystrophin is a cytoskeletal protein encoded by *DMD* (Xp21.1-Xp22), with a central role in maintaining the DGC. The DGC maintains the integrity of the sarcolemma, the plasma membrane of striated muscle tissue. Dystrophin further acts to mitigate contraction-induced injury by functioning as a molecular spring, as well as a molecular scaffold^9,10^. Full-length dystrophin has a molecular weight of 427 kDa, however, due to multiple internal promoters within *DMD*, several naturally truncated isoforms are present, including Dp71^11^.

Accessory proteins have been shown to localise to dystrophin including bona fide mechanotransducers, such as neuronal nitric oxide synthase (nNOS), Yes-associated protein (YAP), and caveolin-3, and therefore represents an important nexus for cell signalling^12–14^. In addition to the adhesome, the cellular machinery associated with cell-matrix interactions, formed by the integrins and their downstream targets, these two complexes represent the critical interface between ‘inside’ and ‘outside’ of the cell. It is essential for cellular behaviour and survival that these focal adhesions are not abnormally disrupted. Moreover, evidence supports dystrophin as a regulator of mechanosensitive ion channels including stretch-activated channels, particularly L-type Ca^2+^ channels and TRPC channels^15^.

Whilst dystrophin is important to the homeostatic function of striated muscle cells, the exact underpinning mechanisms are less obvious, particularly the role of dystrophin and its capacity as a mechanotransducer and mechanoprotector. Several outstanding questions in relation to the absence of dystrophin have arisen, including: alterations in cytoskeletal architecture that may cause changes to the viscoelasticity of the cell, potentially related to its capacity to dampen the forces from the contractions against the ECM^10^; are mechanosensitive proteins mislocalised at the sarcolemma, for example, YAP and AMPK; is there cross-talk with integrins, potentially leading to aberrant mechanotransduction in absence of dystrophin? All of these features may contribute towards the severe DCM phenotype observed in patients with DMD.

Moreover, relating the changes in cellular biomechanics to the overall disease phenotype of DMD is of significant clinical value. DMD is an X-linked muscular dystrophy affecting 1:3500–5000 males and is characterised by the early loss of ambulation (<5 yrs) and progressive DCM, with a significantly poorer prognosis compared to DCM of other aetiologies^16–18^.

The biomechanics of dystrophin loss has not been fully described and here we review the evidence to support the notion that dystrophin does indeed act in a capacity of mechanoprotector- i.e. maintains sarcolemmal integrity- and is critical in mechanotransduction. Moreover, we look at the evidence that suggests an important cross-talk with the integrins, in particular, the laminin-binding α7β1D in striated muscle cells.

### Progressive dilated cardiomyopathy in the context of DMD

Insertions and deletions account for a significant number of mutations in *DMD*, with 72% of all mutations arising from such mutations^19^. Clinically, DMD presents during infancy (≤5 yrs) with patients showing hypotonia, positive Gower’s sign, delayed progression of milestones, intellectual impairment, and skeletal muscle atrophy^8^. Historically, respiratory distress was the leading cause of mortality in patients with DMD, but improved supportive care (corticosteroids, continuous positive airway pressure) has extended the lifespan of these patients with a median age in DMD patients born after 1990 of 28.1 years^20,21^. However, as patient survival has increased, progressive DCM, carrying a significantly poorer prognosis compared to other cardiomyopathies^16^, leading to end-stage heart failure has now become the leading cause of mortality accounting for approximately 50% of deaths in DMD^17,18^.

Progressive DCM is characterised by left ventricular dilatation and increased compliance, thinning of the ventricles, increased fibrofatty infiltration, decreased systolic function, and increased prevalence of arrhythmias^8^. The extent of DCM in patients with DMD is almost ubiquitous by late teens (90% by 18 yrs), but is present in approximately 59% of patients by 10 yrs^8,22^. It is critically important to address this issue as left ventricular ejection fraction steadily declines annually at a rate of 1.6% *per annum*^23^.

Arrhythmias are commonplace in DMD patients, particularly sinus tachycardia and ventricular tachycardia, and are a source of sudden cardiac death^22^. Arrhythmias arise as a result of fibrofatty infiltration, notably in the inferobasal aspect of the left ventricular that disrupts re-entry circuits in conjunction with dysfunctional [Ca^2+^]_i_ handling and dysregulated ion channel function^24,25^. Recognition of the clinical cardiac picture is paramount as earlier therapeutic strategies may delay onset of severe DCM.

Reinforcing the importance of treating cardiac dysfunction as well as skeletal muscle morbidity was shown by an interesting study examining the impact of improving skeletal muscle tissue without addressing the underlying cardiac issues present in DMD, using a DMD murine model termed *mdx*^26^. Here the authors demonstrated a seemingly paradoxical 5-fold increase in cardiac dysfunction in response to skeletal muscle improvement, with mice showing significantly decreased ejection fractions^26^. Improvement in skeletal muscle function enabled higher physical activity that exacerbated the workload on the myocardium rendering it increasingly susceptible to overall dysfunction^26^. This underscores the importance of treating patients with DMD as a whole and cautions against skeletal muscle therapy alone.

### The structure of the dystrophin glycoprotein complex (DGC)

**Fig. 1:**
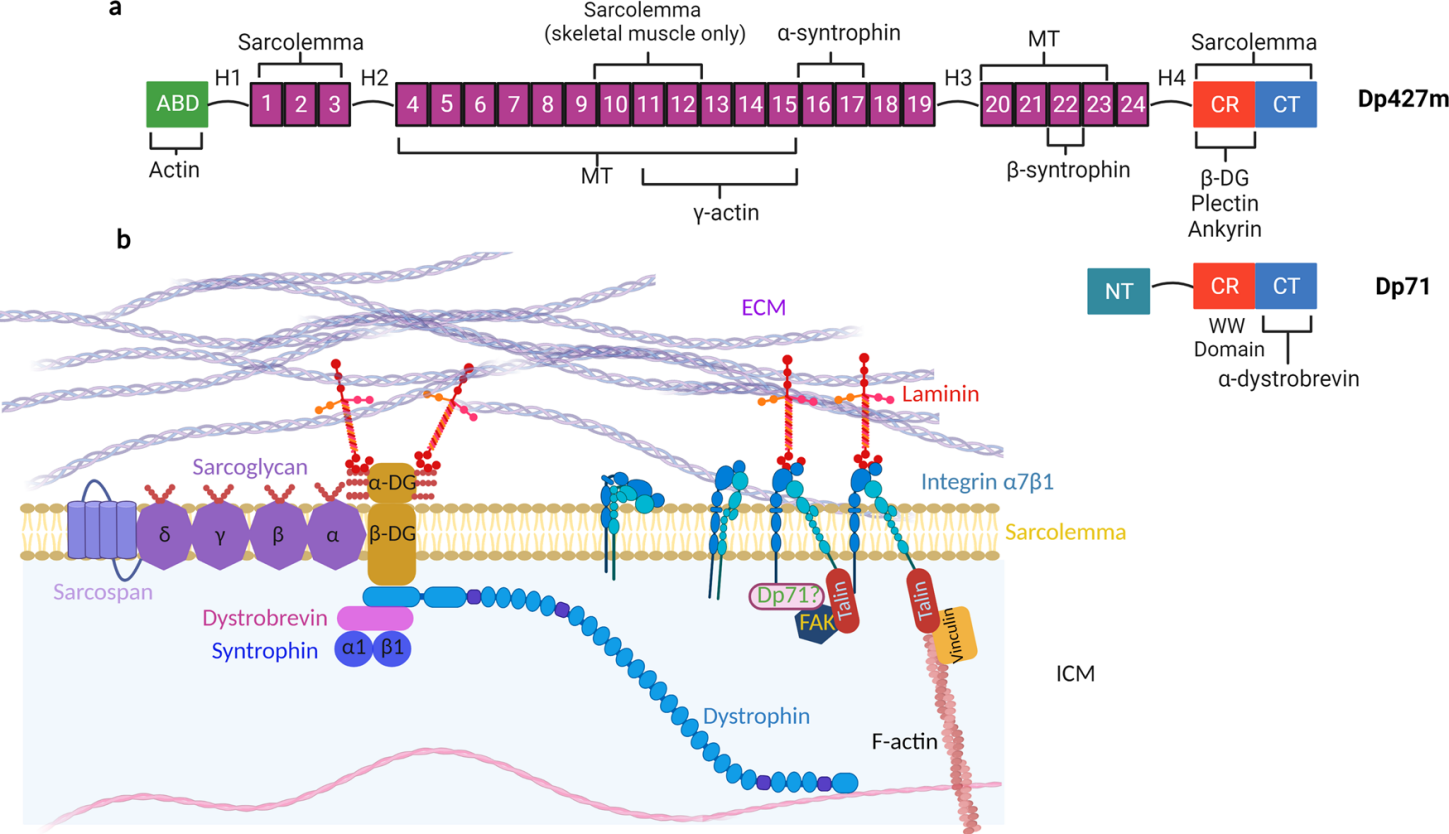
Overview of the Dystrophin Glycoprotein Complex with a Focus on Dystrophin. **a** Schematic of both full-length dystrophin (Dp427m) and the small, truncated isoform, Dp71. Dystrophin has 24 spectrin repeats separated by four hinges, as well as having an actin-binding domain (ABD), cysteine-rich (CR) domain, and c-terminus (CT). Key binding partners are highlighted, including microtubules (MT) and the sarcolemma. There are many isoforms of Dp71, with Dp71m referring to muscle whilst Dp71b refers to neuronal tissue isoforms. Specifically, Dp71f refers to the neuronal cytoplasmic isoform. **b** The dystrophin-glycoprotein complex (DGC) as a whole situated at the sarcolemmal. Biomechanical forces are transduced between the ECM to F-actin. Note the potential cross-talk between the DGC and integrin adhesions, with Dp71 potentially having a role at focal adhesions. Created with Biorender.com.

The DGC has several complementary functions, namely, to provide structural stability to the sarcolemma; be a molecular scaffold functioning as a signalling nexus; regulation of mechanosensitive ion channels; central to mechanotransduction at the costamere; and is associated with lateral force transmission at costameric regions (Fig. 1b). Dystrophin plays the central role in this capacity, and there are several distinct isoforms due to multiple internal promoters, each with distinct roles spanning different tissue. Differential tissue expression of distinct dystrophin isoforms supports the notion of unique roles that each isoform plays. For example, cardiac tissue expresses full length (Dp427m) as well as the shorter dystrophin isoform, Dp71m, whilst skeletal tissue expresses only the former of these two^11^. Looking at the roles that each isoform has may reveal novel insights into not only its physiological function but also the pathogenesis in muscular dystrophies.

DMD is the most prevalent form of muscular dystrophy and is caused by mutations in *DMD*. However, to fully appreciate our current understanding of the role that dystrophin plays, it is important to contextualise it within the entirety of the DGC. Therefore, the other constituent proteins will be briefly outlined. The protein composition of the DGC began to be unravelled during the late 1980s with a particular focus on dystrophin. Seminal discoveries towards the identification of dystrophin were carried out by Koenig^27,28^, Hoffman^29^, and Ervasti^30^ that revealed dystrophin to be a 427 kDa protein in striated muscle tissue^31^.

Subsequently, additional subcomplexes were shown to be associated with dystrophin including sarcoglycans, sarcospans, dystroglycan subcomplex, dystrobrevin, and the syntrophins^8^, together forming the current model of the DGC. This section will first disseminate the evidence for a role of the DGC in mechanosensing whilst looking at the individual components in detail.

### Dystrophin

The full-length dystrophin isoform present in striated muscle tissue is Dp427m (‘m’ indicative of muscle to distinguish from the brain, for example) and is a large rod protein with four functional domains localised at the subsarcolemma in cardiomyocytes, specifically at costameric regions^29,32^. Dp427m is encoded on Xp21.1 by the *DMD* gene and is comprised of 79 exons produced from 2.2 megabases and is thus the largest gene within our genome^8^.

Several internal promoters within *DMD* produce multiple, truncated isoforms of dystrophin, some of which display tissue specificity. In contrast to Dp427m, Dp71m is significantly truncated and does not have the spectrin repeat domains or the N-terminus ABD domain. However, Dp71m does maintain the C-terminal binding structures. In cardiomyocytes, the role of Dp71m is unclear but it has been shown to localise to the T-tubules, indicating that it may serve to regulate excitation-contraction coupling^33–35^. To the best of our knowledge, examination of Dp71m in cardiac tissue has recently not attracted significant attention, but some work has shown it to be involved in stretch-activated ion channels and Masubuchi suggested that it may have a role in the regulation of nNOS^33,36^. That being said, Dp71 has garnered significant attention in neurophysiology and platelet research and these areas may offer insights into a role within cardiomyocytes^37–39^.

Within neural tissue, Dp71b is the predominantly expressed isoform of which 14 sub-isoforms have been reported^38^. Dp71b is an important regulator of aquaporin-4 and Kir4.1 potassium ion channels within the central nervous system, and its absence has been shown to cause alterations in the permeability of the blood-brain barrier^40^. Given the role of Dp71b in regulating ion channels, it is possible that Dp71m is acting similarly in cardiomyocytes.

The presence of the DGC at costameres immediately suggests its role in mechanotransduction and indeed it has been shown to co-localise with integrin-talin-vinculin complexes^41^. Moreover, given that costameres are focal points for lateral mechanical force transduction, the localisation of Dp427m here highlights its role in protecting cells against contraction-induced injury. Downstream, Dp427m interacts with the actin and microtubule cytoskeleton thereby completing the connection of the intracellular *milieu* to the ECM.

Structurally, Dp427m is a filamentous rod protein comprised of four regions (Fig. 1a):

An N-terminus containing an actin-binding domain 1 (ABD1) composed of two calponin homology (CH) domains crucial for the interaction with F-actin and for securing the γ-actin isoform to the sub-sarcolemma^42,43^. By connecting to the sub-sarcolemmal cytoskeleton, dystrophin may contribute to the overall viscoelasticity of cardiomyocytes and its localisation at costameres supports the notion that it is involved in mechanotransduction as well as being mechanoprotective^44,45^.

A central rod domain comprised of 24 spectrin-like repeat proteins, each of which is ~100 amino acid residues in length^8^. The spectrin repeats are interspersed by four hinge domains conferring flexibility and a large degree of extensibility to the protein. The spectrin repeats of dystrophin can extend from 21 nm to 84 nm unfolded within physiological force ranges (15–30 pN), forces achievable by myosin contraction^46^. These features within the spectrin repeats domain allow dystrophin to act as a molecular shock absorber^10^.

The central rod of Dp427m secures its localisation to the sarcolemma, particularly via hydrophobic and electrostatic interactions with phosphatidylserine^47,48^. Interestingly, dystrophin’s central rod interacts with sarcolemmal phospholipids differently between skeletal and cardiac tissue, perhaps reflecting differential ‘spring-’ like modalities^49^. In cardiomyocytes, spectrin repeats R1-R3 and C-terminual/cysteine-rich (CT/CR) domain are crucial whilst skeletal muscle also binds via R10-R12^49^.

Binding to the γ-actin cytoskeleton requires spectrin repeat regions 11–17 of ABD2 composed of basic amino acid residues and is distinct from F-actin binding CH domains. Microtubules directly interact with dystrophin’s rod-domain with spectrin repeat residues 4–15 and 20–23 necessary for this interaction and requiring the presence of ankyrin-B to prevent microtubule loss at this site^50–52^. Disruptions between microtubules and dystrophin have been shown to increase reactive oxygen species (X-ROS) that exacerbates the DMD pathology^53^.

A CR domain that, via ankyrin-B, is another anchor to phospholipids of the sarcolemma^52^. Ankyrin-B and ankyrin-G are required for the costameric localisation of dystrophin/DGC with their absence leading to diffuse sarcolemmal patterning of the DGC^52^.

The CR domain contains a WW binding domain that interacts directly to the PPxY binding motif of β-DG. By connecting to the dystroglycan complex, dystrophin completes the connection between the interior and exterior of the cell^54^. This connection is vital for striated muscle as evidenced by the fact that disrupting the link between the ECM and cell interior causes life-limiting muscular dystrophies.

Lastly, the CT domain is highly conserved region that forms coiled-coils, crucial to binding with α-dystrobrevin and α1-, β1-syntrophins^55,56^. α-dystrobrevin binds to the CT domain of dystrophin providing additional sarcolemmal stabilisation of dystrophin^57^.

#### Utrophin

Utrophin is widely expressed in various tissue including endothelial cells, neuronal tissues, and striated muscle tissue during embryonic and foetal development^58^. Utrophin, expressed by *UTRN* located at chromosome 6q, is the autologous homologue of dystrophin, sharing 80% protein homology. During development, utrophin localises at the sarcolemma though is significantly downregulated postnatally in striated muscle tissues being replaced by dystrophin^56^. Postnatally, utrophin localisation is limited to myotendinous and neuromuscular junctions of skeletal muscle^58,59^.

The binding partners of utrophin are generally similar to that of dystrophin, although some key differences have been described. For example, dystrophin interacts with β-DG specifically through its WW domain that is stabilised by the ZZ domain (named after its ability to bind two zinc ions) within its CT region- with cysteine residues 3307–3354 being particularly important to this interaction^60,61^. Utrophin also binds to β-DG via WW/ZZ domains, but the exact residues underpinning this interaction are distinct to that of dystrophin (3307–3345 in dystrophin vs 3064–3102 in utrophin)^60,61^. Importantly, the binding of utrophin to β-DG was approximately 2-fold lower compared to that of dystrophin^61^. It was reported that dystrophin bound to F-actin via spectrin repeats 11–17 whilst the similar region in utrophin was not able to bind to F-actin, even at high concentrations, but may interact via its CH domain^62–64^. Lastly, unlike dystrophin, utrophin is unable to bind to microtubules^51^.

Biomechanically, the spectrin repeats of utrophin have a distinct unfolding pattern compared to dystrophin^65^. Utrophin spectrin repeats unfold at higher forces similar to that of titin rather than dystrophin^65^. This is consistent with its localisation and role for stiff elastic force transduction at the myotendinous junction but may render utrophin less suitable to act as a molecular spring in the buffering of contraction-induced forces^65^. Together, these data would suggest that there may be altered mechanotransduction and mechanical buffering capacity in the instance of utrophin overexpression, especially in light of differential binding partners/mechanisms, however, this requires further experimental examination.

Functionally, utrophin is considered to perform a similar role to dystrophin, a fact that has made it a target of interest for the potential treatment of DMD^66,67^. In fact, it has been shown that some patients with DMD re-express utrophin, presumably as a compensatory mechanism, and there is success in phenotype rescue in murine models with utrophin overexpression^68^. Whilst upregulation of utrophin is a plausible therapeutic strategy, given the distinction in the form and function of utrophin compared to dystrophin as well as the practicalities of inducing such overexpression with appropriate localisation along the sarcolemma, make long-term utrophin strategies unclear at present. It is interesting to note that female carriers do demonstrate a mosaic pattern of utrophin expression, with the ratio between dystrophin and utrophin potentially impacting the extent of DCM in this class of patients^69^, though murine carrier models have shown comparable cardiac compliance to that of WT^70^ suggesting that mosaics are less affected compared to homozygous patients.

#### The Dystroglycan Sub-Complex

The dystroglycan sub-complex is comprised of two proteins, α- and β-dystroglycan (α-, β-DG) that are both transcribed from the *DAG1* gene, which is then posttranslationally cleaved into the two constituent proteins^71^. α-DG is heavily glycosylated on the extracellular aspect of the DGC and directly interacts with laminin α2 as well as agrin^72^ and pikachurin^73^, and the proline residues of the dystrophin’s CT/CR region^73–76^. The *O*-linked glycosylation, particularly that of serine residues, is essential for its interaction with the ECM. The glycosylation pathway involves several enzymes, mutations of which cause muscular dystrophies (see also Table 1). These include  the O-mannosyltransferase POMT2, fukutin and fukutin-related protein (FKRP), both ribitol-phosphate transferases that add a tandem ribitol phosphate to the core glycan, as well as the protein LARGE1, which adds a linear polysaccaride of xylose and glucuronate, also called matriglycan onto the end of the glycan^77^. FKRP is also involved in the development and maintenance of the ECM with mutations leading to decreased laminin α2 and α-DG expression^77–79^. Moreover, FKRP may also direct basal lamina formation and cardiac ECM by glycosylating fibronectin^80^.

β-DG contains a PPxY binding motif that directly localises, and sequesters, YAP^12^. This was an interesting revelation as it implicates the DGC in regulating the cell cycle of cardiomyocytes. α-DG in neonatal cardiomyocytes interacts with agrin which promotes cardiac regeneration at the expense of cell maturation as well as promoting dissolution of the DGC^76^. As cardiomyocytes mature, agrin expression decreases in favour of laminin, which is thought to promote cell-cycle arrest^76^. Morikawa^12^ went on to show that double knockouts of dystrophin and Salvador (a negative regulator of YAP) led to over-proliferation of cardiomyocytes at a scar generated by an infarct. This has led to the exciting notion that manipulation of YAP could be clinically valuable against tissue loss postmyocardial infarction. Dissolution of the DGC induced by agrin may therefore represent an axis that permits the activation of YAP and is a potential avenue for cardiac regeneration.

Mechanically, α- and β-DG are required to maintain the interaction between the sarcolemma and the basal lamina^81^. Both α-DG and α7 integrin contribute to force production at costameres, the absence of α-DG causes dissociation of the sarcolemma from the basal lamina^81^, rendering skeletal muscle tissue susceptible to contraction-induced injury. As mentioned previously, the dystroglycan complex regulates the overall turnover of the DGC where engagement with the cognate ligand laminin resulting in tyrosine phosphorylation 892 of β-DG’s PPPY binding motif^82^. Tyrosine phosphorylation here promotes disassembly from dystrophin, allowing the DGC complex to be turned over. Physiologically, this process is highly regulated, a feature that is lost in muscular dystrophies^82^, though the underlying mechanisms governing this process are not fully understood.

Cyclic stretch has been shown to activate ERK1/2 and AMPK pathways via the dystroglycan complex and the associated protein, plectin^83^. Together, plectin and dystroglycan were required to act as not only a scaffold but are involved in mechanotransduction, with the knockdown of plectin leading to decreased ERK1/2 and AMPK activity^83^. Plectin also binds to the cytoskeletalintermediate filament desmin whose overexpression was shown to ameliorate the disease phenotype in the DMD murine double knockout model *mdx:desmin* and *mdx* mice^84^. By interacting with β-DG, plectin connects the DGC indirectly to this component of the cytoskeleton. Moreover, dystroglycan interacts with the growth factor receptor-bound protein 2 (Grb2) which is known to be involved with cytoskeletal rearrangement^85^. Integrin activation of Ras was shown to be mediated via Grb2 and this may present a potential avenue for cross-talk between integrins and the DGC^86^.

Mutations of genes involved in the glycosylation of α-DG result in the so-called dystroglycanopathies. Dystroglycanopathies display clinical heterogeneity but are all fundamentally caused by disrupting the interaction between α-DG and laminin α2^77^. Dystroglycanopathies caused by primary mutations in *DAG1* are, generally, extremely rare, likely due to them being embryonically lethal^87^, underpinning the necessity for the cells binding to the ECM. This means that the majority of dystroglycanopathies are caused by secondary mutations in proteins associated with glycosylation. For example, mutations in *POMT1* give rise to the extremely severe Walker-Warburg Syndrome, characterised by lissencephaly and a significantly reduced lifespan of less than 3 years^88^. However, mutations in *FKRP* largely manifest as Limb Girdle Muscular Dystrophy (LGMDs), which are often, though not always, relatively mild. Mutations in *FKRP* have, however, been shown to be a rare cause for WWS^89^. Numerous mutations have been identified in *FKRP* with the founder mutation (c.826>A) most commonly causing LGMD2I^90^.

LGMD2I is a relatively mild form of muscular dystrophy and underpinning its pathogenesis is the disruption between the ECM to the intracellular cytoskeleton. What is less clear is the relationship between the genotype and phenotype in patients with mutations in these genes, and indeed this concept applies to other proteins of the DGC. Why do some patients with mutations in *FKRP* exhibit a disease phenotype consistent with WWS whilst others have LGMD2I? The answer to this may lie in i) which stage of the glycosylation pathway the mutation disrupts, or ii) the extent of the hypoglycosylation for any given stage. Hypoglycosylation of α-DG may still permit a degree of interaction to the ECM, resulting in a milder phenotype overall, whilst dissociation from the basal lamina increases the severity in disease phenotype. LGMD2I patients also develop DCM although this is less well documented, compared to DMD, thereby motivating an urgency to understand these mutations in the context of cardiomyocytes.

#### The sarcospan-sarcoglycan sub-complex

The sarcospan-sarcoglycan sub-complex contributes to the formation of the DGC and directly interacts with β-DG. In cardiac tissue, four single-pass sarcoglycans are present: α, β, γ, and δ^91^. Recently, a missense mutation c.218C>T in exon 3 as well as a partial heterozygous deletion in exons 7–8 within the *SGCA* gene was described as causing LGMD2D^92^. However, the authors did not assess the cardiac phenotype in this case.

Other groups have identified *SGCD* in both porcine^93^ and murine^94^ models that lead to a decreased expression of proteins in the sarcoglycan sub-complex thereby disrupting the overall structure of the DGC and resulting in DCM. Moreover, it has been reported that 19% of all patients containing a mutation of *SGCA, SGCB*, or *SGCG* displayed DCM with 25% of all patients also requiring respiratory support^95^.

Recessive mutations in sarcoglycan (SG) δ lead to the reduction or complete absence of the sarcoglycan complex, and subsequently the DGC, in myocardial tissue and are a cause for LGMD with associated DCM^96^. Interestingly, dominant negative mutations in SG-δ are cardiovascular specific and are a cause for familial dilated cardiomyopathy^97^. The SG-δ dominant negative mutations R97Q and R71T were shown to be stably expressed in rat cardiomyocytes without causing significant disruption to the overall DGC^98^. However, under mechanical stress cardiac cells harbouring these mutations were more susceptible to sarcolemmal damage and permeability as well as mechanical dysfunction, consistent with a DCM phenotype^98^.

Sarcospan (SSPN) is a 25 kDa tetraspanin protein that localises with the sarcoglycan sub-complex and is thought to act as a protein scaffold^99,100^. In its role as a protein scaffold, SSPN stabilises the localisation and glycosylation of α-DG^99,101^. Overexpression of SSPN in murine models was revealed to increase binding between muscle and laminin^102^. Furthermore, SSPN has also been shown to interact with integrins, which provides evidence that there is a degree of crosstalk between both costameric focal adhesion sites, the DGC, and the integrin-talin-vinculin glycoprotein structures^100–102^. Knockdown of SSPN also led to an increase of α7β1 in murine skeletal muscle.

A recent study showed that overexpression of sarcospan enhanced the maturation and glycosylation of α-DG in cardiac tissue independently from galactosaminyltransferase 2 (*Galgt2)* knockdown in *mdx* murine DMD model, thereby alleviating the disease phenotype^101^. Increased glycosylation of the dystroglycan complex may strengthen the interaction to the ECM therefore mitigating the disease. Moreover, they showed that sarcospan overexpression decreased the interaction of integrin β1D with the DGC, highlighting a possible role for sarcospan in regulating integrin complexes^101^.

### The syntrophins

The Syntrophins are a family of small (58kDa) proteins localised to the DGC with no intrinsic enzymatic activity of their own that act as molecular adaptors^103,104^. Five isoforms have been identified (α-1, β-1, β-2, γ-1, and γ-2), demonstrating tissue-specific expression, with the α-1 isoform being predominantly expressed by striated muscle tissues^105^. Syntrophins are important adaptor proteins that promote association between dystrophin and signalling molecules, including neuronal nitric oxide synthase (nNOS) in skeletal muscle^106^. α-syntrophin directly interacts with dystrophin’s spectrin repeats 16–17 domain that in turn binds to the PDZ binding motif of nNOS^106,107^.

Syntrophins also interact with dystrobrevin, via PH2 and SU binding domains, and these also interact with the actin cytoskeleton^108^. Indeed, the syntrophins appear to have a particularly pivotal role in modulating cytoskeletal dynamics with the α and β isoforms being able to directly interact with F-actin^108^, and may therefore have a role in regulating the tensegrity and biomechanics of the cell. Furthermore, syntrophins have been shown to modulate the cytoskeleton via Rac1^109^.

Modulation of syntrophin levels can restore functionality as recently demonstrated by a study looked into using micro-dystrophin and found that ΔR4-R23/ΔCT construct was able to restore α-syntrophin-as well as other DGC proteins- to levels comparable to WT in an *mdx* cardiomyocytes^110^.

In addition to their role in modulating the cytoskeleton, the syntrophins are well documented in ion channel regulation^111–113^. The PDZ binding-motif of the syntrophins regulates the cardiac voltage-gated channel, Na_v_1.5^111^, which has a pivotal role in establishing cardiac excitability and conduction. Interestingly, in *mdx* murine models Na_v_1.5 channel was found to be downregulated, with animals displaying arrhythmias^111^. Moreover, the mechanosensitive ion channel family, the transient receptor potential channels (TRPC) have also been shown to be under the regulation of α1-syntrophin in cardiac tissue^113^ with TRPC6 inhibition ameliorating arrhythmia in a murine model of DMD^112^. Increased activity of TRPC6 was reported in DMD causing arrhythmia that was reversed when PKG was bound^112^. Mechanistically, the absence of dystrophin promotes stretch-induced [Ca^2+^]i influx which acts upstream of TRPC6, thereby activating it with studies demonstrating this in cardiomyocytes and vascular smooth muscle cells^112,114^. The hyperactivation of TRPC6 in response to stretch makes it a primary mechanosensor and potential therapeutic target in DMD^112,114^.

#### The entire DGC complex functions synergistically: the sum is greater than the individual components

The absence of dystrophin can cause dissolution, or significant downregulation, of the entire DGC complex and many of the mechanoprotective and mechanotransductive features are subsequently lost, resulting in the catastrophic phenotype observed in striated muscle tissue in DMD. As such, it is perhaps prudent to consider that the DGC works synergistically, and that the individual constituents rely upon the presence and function of the others. This holds particularly true for dystrophin which seems essential in the assembly and localisation of the complex at the sarcolemma in cardiomyocytes. Each of the constituents has their own unique role that contributes to the overall stabilisation of the sarcolemma, localisation of key accessory proteins, regulation of ion channels, and gene expression with the deletion of one protein of the DGC rendering the entire myocardium dysfunctional.

#### The DGC is well positioned to act as a bona fide mechanotransducer and is critical to cardiomyocyte health

As shown above, many of the proteins of the DGC are involved in mechanotransduction and signalling, with dystrophin being particularly primed for this role. Provided that the DGC is located at costameres supports the notion that it is involved in mechanotransduction alongside the integrins. Therefore, the DGC is physically receptive to anisotropic force transmission, and is involved in the in cell’s mechanosensing of the microenvironment and cytoskeletal rearrangement, consistent with the tensegrity model^115^. Moreover, Dp427m acts as a mechanoprotector by buffering incoming biomechanical forces by extending spectrin repeats within its central rod domain maintaining unravelling forces at 25 pN over an 800 nm extension^10^. By unravelling, dystrophin is able to ‘buffer’ against contraction-relaxation forces generated by the cardiomyocytes^10^. Given the diversity of proteins and phospholipids that interact with the spectrin repeat domain, it is interesting to speculate whether unravelling of the spectrin repeats alters the binding kinetics of mechanosensitive proteins, in a manner analogous to that of talin^116–118^. However, this is currently not established and requires further examination.

The N-terminus of dystrophin directly interacts with the γ-actin and F-actin cytoskeleton^43,119^, and therefore biophysical forces may be transmitted via this region to the intracellular matrix (ICM) via this route, again consistent with tensegrity and may then regulate cytoskeletal architecture and dynamics. Additionally, the cytoskeleton may then propagate these forces to the nucleus via the linker of nucleoskeleton and cytoskeleton (LINC) complex^120^. Studies have revealed that mechanotransduction can be more than 40 times more rapid compared to soluble signalling, that in turn regulate chromatin and gene expression apparatus^120–124^. Dystrophin interacts with microtubules, and it is the interaction of both of these that makes dystrophin well poised to play a critical role in the tensegrity of cardiomyocytes^51,125^. Indeed, it has been shown that dystrophin is essential to the maintenance of γ-actin and microtubule lattice formation at the sub-sarcolemma^43,125^.

There is tentative evidence to suggest a link between cytoskeletal dynamics and nuclear mechanotransduction in DMD. Recently, a study revealed a link between gene regulation and the cytoskeleton in DMD^126^. Here, they showed the deleterious upregulation of histone deacetylase 8 (HDAC8), that they selectively inhibited, improving skeletal muscle function. Interestingly, inhibition of HDAC8 led to increased acetylation of α-tubulin that restored cytoskeletal architecture in the myotubes of DMD patients^126^.

Lastly, accessory proteins localise at the DGC, including ERK1/2, Grb, and nNOS, and some are particularly receptive to mechanotransduction, such as AMPK^13^. Dystrophin is thought to interact with AMPK via intermediate proteins, including sarcolemmal dysferlin^127^. Interestingly, unlike skeletal muscle, nNOS does not directly localise with the DGC in cardiac tissue, though is phosphorylated in response to AMPK mechanical activation. This occurs in response to stretch to form a dystrophin-AMPK-nNOS axis^13^ and as mentioned previously interacts directly with α-syntrophin^106^. In *mdx* model the dystrophin-AMPK-nNOS axis was disrupted and could be restored pharmacologically, indicating that force was the primary driver for nNOS activation in cardiomyocytes- the drug bypassed the need for force. These data support the notion that dystrophin, and the DGC as a whole, acts in response to force to upregulate accessory proteins, connect to the cytoskeleton, and contribute to the overall viscoelasticity of the cardiomyocyte.

#### Cross-talk between integrins and the DGC

Integrins are a superfamily of transmembrane proteins responsible for focal adhesion formation, mechanosensing of the ECM, and mechanotransduction^1^. There are 24 distinct integrins formed as heterodimers from 18 α- and 8 β- subunits that display tissue-specific expression patterns^128,129^. Moreover, integrin expression within the same tissue is subject to spatiotemporal changes, for example in cardiomyocytes a shift occurs from fibronectin integrins embryonically, e.g. α5β1, towards the laminin-binding α7β1D integrin as the tissue matures^130,131^.

In cardiomyocytes, the integrins localise at costameric regions and are involved in mechanotransduction, regulation of the actin cytoskeleton, and governing cellular processes (e.g. migration). Importantly, given their interaction to the cytoskeleton, they are heavily involved with maintaining the viscoelasticity of the cell as described by the tensegrity model^132–134^. It is beyond the scope of this review to detail integrin activation and specific downstream targets, however, the following references permit further exploration of this area^1,128,135,136^. In brief, the integrins are capable of rigidity sensing where an increase of force across the ECM strengthens the bond between the integrin (as well as recruitment of additional integrin units) and the ECM, a behaviour called a catch-bond^137^. Integrins have been widely described in the context of focal adhesion formation and maturation, allowing cells to sense their microenvironment and assess ECM rigidity and communicating this biomechanical ‘information’ along prestressed actin cables^122,138,139^.

Evidence supports cross-talk between the DGC and the integrins, which may not be particularly surprising given their similar locale and synergistic functions^41,140,141^. Mainly, these insights derive from studies using mutations in either the DGC or integrin proteins causing various muscular dystrophies; double knockout studies revealing more severe disease phenotype than individual knockouts i.e. accelerated myopathy and premature lethality; and compensatory expression of integrins in the absence of dystrophin^5,142,143^. Together, these three facts strongly suggest cross-talk and co-regulation.

Previous work has shown that mutations in the integrin α7 (*ITGA7)* were a cause of congenital muscular dystrophy with a disease phenotype not entirely distinct to DMD^6,144^. Here, Mayer^144^ showed that the absence of α7 caused necrosis of myofibres, centralised nuclei, and disrupted sarcomeric architecture all consistent with a later finding in humans harbouring primary mutations in *ITGA7*^145^. In this instance, interestingly, the DGC did not appear to compensate for the loss of α7^144^.

On the other hand, the absence of dystrophin – as in DMD or the *mdx* model – has consistently revealed an upregulation in the α7 integrin, thought to be a compensatory mechanism, although this compensation appears insufficient long-term in DMD patients^144–147^. That being said, overexpression of α7 pharmacologically or adeno-associated viruses delivery has shown attenuation of the DMD disease phenotype in both skeletal and cardiac tissue^148^.

Of particular promise is sunitinib, an FDA-approved tyrosine kinase inhibitor, that increases expression of α7β1, mitigating cardiac fibrosis, improving attachment to the basement membrane, and decreasing STAT3 - a promoter of cardiac fibrosis^143^. A similar compound has also been used, SU9516 that broadly led to similar outcomes as sunitinib^149^. Interestingly, the glucocorticosteroid prednisone, a standard component of DMD therapy, also increases α7β1 in skeletal muscle of patients with DMD and the golden retriever model, GRMD^150^. However, it was not fully determined if this mechanism functions similarly in cardiac tissue.

Conversely, overexpression of sarcospan has been shown to upregulate β1D expression in cardiac tissue in a dystrophin-utrophin double knockout *mdx:utn*^*+/-*^ model that concomitantly improved sarcolemmal stability, lending itself in support of cross-talk^151^. Lastly, a *dag1* knockout modelling dystroglycanopathy showed elevated α7 expression that was able to attenuate, but not fully rescue, the healthy phenotype^152^. Here, the authors suggested that compensation was beneficial in attenuating the disease phenotype but was insufficient for long-term disease prevention, an observation consistent with DMD patients^152^. The evidence provided does suggest that there is a degree of cross-talk with complementary, synergistic, functions between the integrins and the DGC.

The short isoform, Dp71f, has reportedly been shown to directly interact with β1 integrin in neuronal tissue as well as downstream mechanotransducive proteins including FAK^153^. Moreover, Dp71f localised at focal adhesions in astrocytes and co-immunoprecipitated with β1 integrin as well as vinculin and actinin^154^. To the best of our knowledge, this has not been demonstrated in cardiomyocytes, but these studies offer a glimpse into a putative role for Dp71-integrin cross-talk in cardiomyocytes.

Other studies have shown that double knockout of integrins and DGC components – such as dystrophin or sarcospan – result in an exacerbated, rapidly advancing disease phenotype than either knockout alone, suggesting that the compensation by one or other group does indeed attenuate disease phenotype^102,148,151^. For example, the *mdx:β1* double knockout showed worsening cardiac dysfunction that more rapidly progressed towards a heart failure phenotype compared to knockout of *mdx* or *β1* alone^142^.

Overall, these data indicate that not only do the DGC and integrin complexes co-localise, but that there is complementary, synergistic compensation occurring when either is disrupted. This stands to reason as facilitating appropriate attachment to the basal lamina of the ECM as well as maintaining mechanotransduction and sarcolemmal integrity have been shown to be necessary for optimal striated tissue muscle health. However, it is clear from the pathogenesis in patients that compensation over time is insufficient to stave off end-stage cardiac failure, but perhaps pharmacological manipulation of these complexes may be able to delay the onset of significant pathology. In any case, this is a promising area that demands further exploration to offer more therapeutic strategies to patients with diverse muscular dystrophies.

To understand the function of the surface cell receptors, a discussion of the relevant substrate to which they bind is necessary. The cardiac extracellular matrix (ECM) is a diverse, plastic, three-dimensional, structural meshwork that maintains the geometry of the heart^155^. Embedded within this network are cardiomyocytes, cardiac fibroblasts, endothelial cells, and resident macrophages, that all contribute to the overall homeostasis of the heart. Far from being an inert and passive entity, the ECM is intimately involved with regulating cardiomyocyte functions including force transmission, cytoskeleton dynamics, proliferation, as well as acting as a reservoir of cytokines, metalloproteinases, and other signalling proteins^155,156^. The ECM responds to cardiomyocyte biochemical and biomechanical actions and, in tandem, promotes a spatiotemporally regulated matrix optimally suited to housing cardiomyocytes. At the organ scale this translates to a functional heart able to perform its diastolic and systolic functions that are essential for life.

### The cardiac extracellular matrix is critical to cardiac (patho)physiology

#### The healthy cardiac extracellular matrix

The biochemical and biomechanical profile of the cardiac ECM alters throughout development and disease^156^. The embryonic cardiac ECM expresses collagen I, chondroitin sulfate, fibulin, and fibronectin, amongst other constituents^155^. Fibronectin is instrumental in orchestrating the initial myocardial developmental steps by promoting cell migration, adhesion, and polarity^156^ in particular the expression of the embryonic isoforms EIIIA and EIIIB^157,158^. Expression of fibronectin is critical to the development of the nascent heart and mutations have been shown to be embryonically lethal, underpinning the crucial role of the cardiac ECM^159,160^.

A phenotypic switch of the predominant proteoglycans occurs in myocardial ECM with the glycosylation of α-DG marking the transition from nascent/embryonic fibronectin-rich cardiac ECM towards the laminin-211 binding mature cardiac ECM^80^. This process also establishes a DGC-ECM axis, establishing maturation of the costameric and focal adhesion regions that allow mechanotransduction^80^.

In addition to laminin, the adult heart expresses collagen I (80%) and collagen III (10%) with the ratio between these two collagens being particularly important^161^. Type I collagen is a determinant of tensile strength and stiffness, whilst type III collagen confers elasticity to the ECM. Both contribute to the overall viscoelasticity of the ECM to give a Young’s modulus of ~10 kPa^162^. The remainder of the cardiac ECM is comprised of several glycosaminoglycans and proteoglycans^156^.

Given the importance of the interaction between the DGC and laminin, more detail on the laminins is provided. The laminins are a large family of heterotrimeric proteins composed of three peptide chains (α, β, and γ) located in the basement membrane compartment of the cardiac ECM, with key functions in cell adhesion, mechanotransduction, and cross-linking other proteins^161,163^. Laminin α2 is a particularly important cardiac ECM constituent in the context of muscular dystrophies as it is the direct binding partner to the DGC, specifically via α-DG as well as engaging the highly expressed cardiac integrin, α7β1D^164^. Severing the interaction between the DGC and laminin α2 causes the phenotypes observed in Duchenne Muscular Dystrophy (DMD), Becker Muscular Dystrophy (BMD), and Limb Girdle Muscular Dystrophy Type 2I (LGMD2I)^8,165,166^. As mentioned previously, mutations in the *ITGA7* gene that encodes for α7β1D causes a congenital muscular dystrophy with a phenotype similar to DMD^5,144^.

Although the exact mutations causing disruption between ECM and the cell interior differ, the overall concept is that disrupting the connection promotes muscular dystrophy in striated muscle tissues. By breaking the link to the ECM, biomechanical forces are lost, thereby disrupting key downstream mechanotransduction and mechanical cues. Moreover, the loss of this connection is sufficient to disrupt the cytoskeleton, decreasing its responsiveness to ECM mechanics^167^.

#### The cardiac ECM in DMD associated DCM

Whilst it is true that adult cardiomyocytes are able to proliferate to a small degree (a rate of 1% *per annum*) this is generally considered to be insufficient to replace any lost tissue *en masse*, thereby making heart failure a leading cause of morbidity and mortality globally^168^. Moreover, patients with DMD and other muscular dystrophies develop progressive DCM, which is now the leading cause of death within the category of muscular dystrophy diseases^18^. Therefore, it is important to consider how alterations to the cardiac ECM may impact cardiomyocyte homeostasis and vice versa, to elucidate the underpinning mechanisms that drive the pathogenesis of muscular dystrophies.

In response to injurious stimuli, ageing, and disease, the cardiac ECM undergoes expansion in a process termed remodelling^156,169^. Remodelling results in alterations to the biochemical and biomechanical composition of the ECM, often exacerbating any underlaying pathology^170^. In this way, the cardiomyocyte-ECM interaction results in a positive feedback axis driving the pathogenesis towards heart failure. It is worth bearing in mind that heart failure is a diverse, heterogenous disease state with many, distinct aetiologies. Therefore, the specific interaction between the ECM and cardiomyocytes in the context of DMD is of considerable interest, especially as fibrosis, a hallmark of DMD, has been shown to correlate with poor clinical outcomes^22,171^.

The initial phases of remodelling involve the activation of proinflammatory and profibrogenic cytokines, including Transforming Growth Factor (TGF) β, and lysyl oxidases (LOX) that normally reside within the ECM^171^. In turn, these promote increased deposition of collagen type I, thereby altering the ratio between collagen I and collagen III, thus increasing the stiffness of the ECM^155,172^. Increased deposition of cross-linked collagen type I promotes diastolic dysfunction as the increased stiffness of the ECM leads to decreased compliance of the heart^173^, as well as promoting arrhythmias by disrupting re-entry circuits^174^. Moreover, alternatively spliced isoforms of fibronectin, such as type III repeat extra domain A (EDA), are expressed promoting the recruitment of myofibroblasts and monocytes leading to increased cardiac fibrosis^175–177^. Inhibition of fibronectin overexpression was shown to attenuate fibrosis associated with heart failure and improved cardiac function for 4 weeks post-ischaemia^175^. TGF-β was also found to decrease the activity of matrix metalloproteinases whilst concomitantly increasing the expression of profibrotic enzymes, such as tissue inhibitors of metalloproteinases^178^. Lastly, fibronectin EDA also promotes the activation of nuclear factor κB (NFκB), that together promote adverse cardiac remodelling^176^. Altogether, these data highlight the important and dynamic role that the cardiac ECM plays in maintaining appropriate biomechanics and in muscular dystrophies the interplay between cells and ECM is a central driver of the pathogenesis.

#### Cardiac ECM mechanics

The ECM undergoes significant alterations in DMD including fibrosis, elevated inflammatory infiltrate, and cardiomyocyte necrosis, and therefore in this section we look at these changes. As the cardiac ECM has a critical role in maintaining physiological force transmission at the cellular level, as well as regulating diastolic and systolic function, any alterations to the biomechanical profile can distort mechanotransduction^155^.

Viscoelasticity in healthy myocardium is reported as ~10 kPa^179^ increasing to as much as 50–130 kPa in the fibrotic myocardium^162,169^. This is a direct consequence of the increased deposition of collagen type I and fibronectin^155,158,175^, with several groups reporting increased stiffness in diseased hearts^173,180–182^. Increased substrate stiffness leads to abnormal cell morphology, disrupted sarcomeric architecture, abnormal electromechanical coupling, aberrant mechanotransduction, posttranslational modifications of the cytoskeleton-notably microtubules, and altered gene expression^173,183–185^.

The mechanosensing apparatus of cardiomyocytes at costameric regions allows bidirectional communication via a cytoskeleton-DGC-ECM axis alongside the integrins, and therefore influences substrate rigidity^169,186^. Cardiac passive stiffness is determined by the interaction of titin and microtubule network to the sarcomere^169^ with the posttranslational modification de-tyrosination mediating this interaction^187,188^. De-tyrosinated microtubules are increased in DMD and is a driver of cardiac pathogenesis by promoting X-ROS, altered cell stiffness, and dysregulated Ca^2+^ handling^53,189,190^. Decreased de-tyrosination of microtubules in *mdx* model improved the overall cardiac function, as determined by decreased contraction-induced injury and incidence of arrhythmias, highlighting the importance of the cytoskeleton^53^.

The interplay between disrupted DGC leading to altered mechanosensing with cardiac fibrosis, that in turn leads to further alterations in mechanosensing is less well defined, but it is known that DMD exhibits altered biomechanical responses^53^. Remodelling of the myocardium has shown itself to be a cause for necroptosis via the activation of receptor-interacting kinase 3 (RIPK3)^191^. Activation of RIPK3 was shown in skeletal muscle in an *mdx* model, in line with the previous study^192^. Recently it was shown that myocardial fibrosis activated a RIPK1-RIPK3 complex that promoted cardiac dysfunction and decreased autophagy, leading to increased cell death^193^. Moreover, fibrotic stiffness can have a deleterious impact on the genome of DMD cardiomyocytes, where the increased stiffness can drive shortening of the telomeres as well as promoting activation of p53 and p21^194^.

### The role of the DGC in cardiomyocyte mechanotransduction

#### Overview of mechanotransduction

**Fig. 2:**
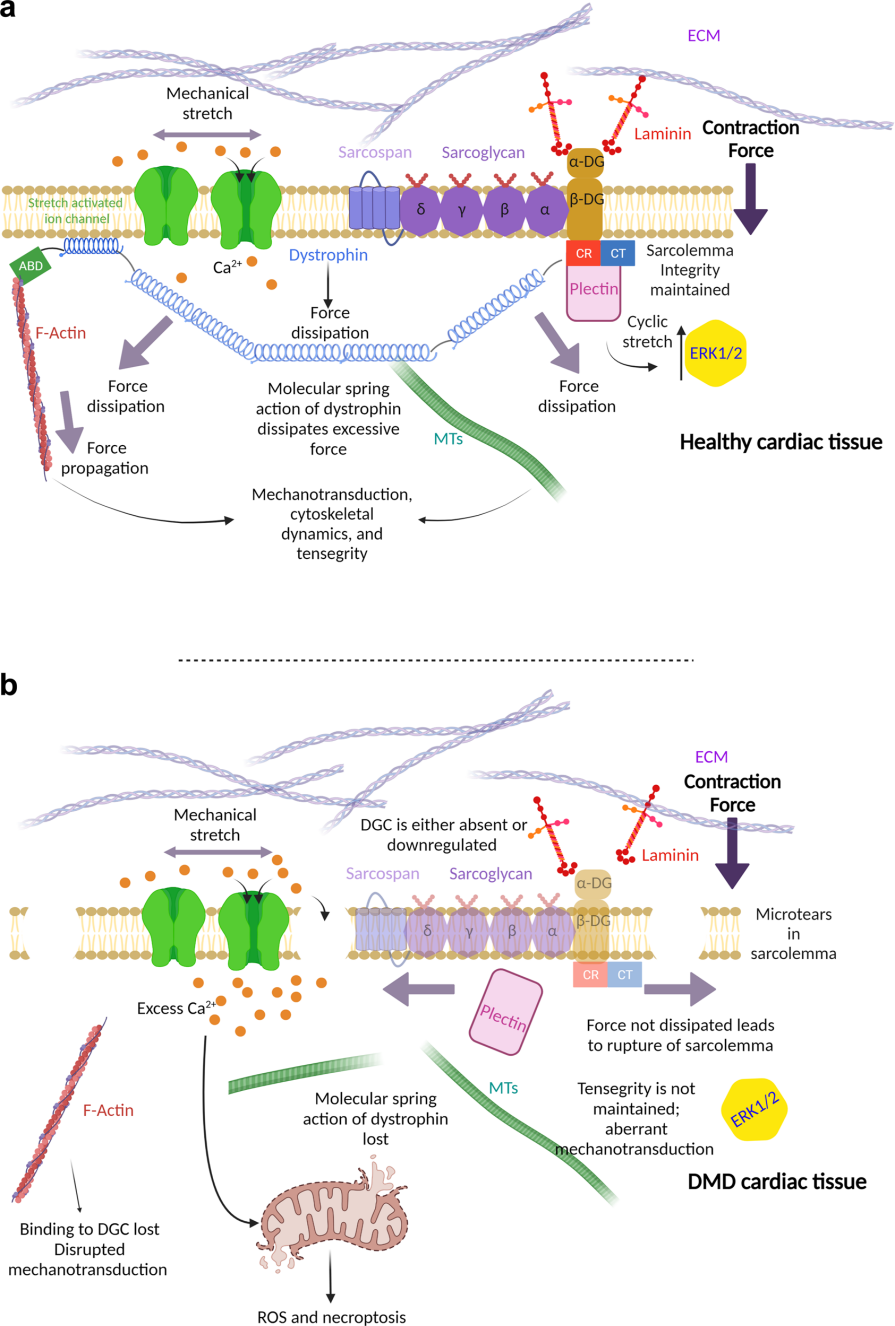
The dystrophin-glycoprotein complex has a central role in biomechanics. **a** Dystrophin is central to mechanotransduction in healthy cardiac tissue. Biomechanical force is propagated along pre-tensed actin and microtubule (MT) cables which can then be transmitted to the nucleus. Moreover, this mechanism allows the cardiomyocyte to maintain tensegrity and respond to changes in the ECM and is perhaps involve in rigidity sensing. Stretch activated ion channels are regulated by dystrophin mediating appropriate Ca^2+^ ion entry, important for excitation-contraction coupling as well as signalling. Plectin associates with β-DG and is regulates ERK1/2 activity. **b** In DMD cardiac tissue, the absence of dystrophin leads to contraction-induced microtears of the sarcolemma, allowing excess entry of Ca^2+^ ions, leading to mitochondrial dysfunction and cell death. Moreover, the biomechanical signals are no longer propagated along actin and MT cables causing aberrant mechanotransduction. In the absence of dystrophin, the whole DGC can become absent or is heavily downregulated causing further disruption to downstream signalling. Created with Biorender.com.

Whilst biochemical and genetic cues have long been known as regulators of cellular biology, it is increasingly appreciated that so too are physical forces. Cells are responsive to biomechanical force with roles in differentiation, embryogenesis, focal adhesion formation, cell migration, proliferation, survival, cell morphology, and gene regulation, and the DGC is central to this process (Fig. 2). The cytoskeleton is central in maintaining the viscoelasticity of the cell and for the bidirectional communication of biophysical force between the ECM and ICM of the cell. Alterations of the cytoskeleton, and subsequently cellular viscoelasticity, in response to biophysical cues is a key determinant for maintaining cellular homeostasis, with disruptions in the ICM-ECM connection affecting these processes. An exciting area of research will be integrating mechanical cues to chemical and genetic changes that lead into higher order cellular responses; how do these forces influence cell behaviour and ‘decision making’? Currently, these questions remain elusive.

#### The DGC plays a role in maintaining the tensegrity of cardiomyocytes

Tensegrity is a model describing how prestressed structures are able to physically support themselves using a connected system of compressive and tensile elements^195^. The forces on the compressive and tensile elements are in equilibrium with external force application causing remodelling of the structure in order to maintain force equilibrium.

For cardiomyocytes, tensegrity describes how a prestressed cytoskeleton is well poised to transduce and propagate mechanical forces into the cell. Tension is generated by the actin cytoskeleton whilst the microtubule network forms the compressive elements of the system^115,195^. The balance between tensile and compressive elements results in the overall prestressed state of the cell and is the significant contributor to the overall viscoelasticity of the cardiomyocyte. As such, alterations of the cytoskeleton, in particular cortical actin which is a key regulator of cell surface tension, can be assessed by measuring Young’s modulus of the cell^196^.

The prestressed state of cells makes them responsive to external changes of mechanical force that cause a rearrangement and redistribution of the cytoskeletal components. To adapt to the new mechanical force involves the formation of actin cables as well as microtubule buckling and reformation^115^. The propagation of forces along prestressed actin cables has been shown to act more than 40-fold more rapidly than soluble ligand interactions^120,122^, indicating that mechanical force influences cell behaviour acutely and can do so over longer time-scales. Therefore, this mechanism allows cardiomyocytes to sense changes in the ECM, for example, alterations in ECM stiffness, and mount an appropriate and rapid cellular response to it.

Cells respond to alterations in the composition of the ECM by adapting to mechanical force changes, cellular viscoelasticity, cell migration^197^, proliferation^197^, differentiation^198^, focal adhesion formation^199^, as well as being central to driving disease pathologies. Recently, dystrophin-deficient C2C12 myoblasts showed significantly disrupted focal adhesion and altered YAP localisation in these cells, showing that dystrophin has a vital role in mechanotransduction and communicating the ECM to the intracellular *milieu*^200^. Indeed, focal adhesion disruption renders the cytoskeleton-DGC-ECM axis dysfunctional and is sufficient in leading to cardiac dilatation and increased compliance in *mdx* models^70^. Interestingly, in female carrier *mdx*, where 50% of the cardiomyocytes express a functional DGC, did not show this increased compliance in response to mechanical stretch^70^. The authors demonstrated that this effect was distinct from sarcolemmal damage and was linked to the disruption between cardiomyocyte-ECM by using knockouts of other costameric region proteins, including β-SG^70^.

The DGC is a key focal point for lateral force transmission in striated muscle tissue across the costameres, connecting sarcomeres downstream via the Z-discs^41^. The sarcomere-ECM connection allows communication and transduction of mechanical forces into biochemical and genetic alterations, ultimately governing striated muscle behaviour. One study showed, using magnetic micromanipulation, that disruption of the actin cytoskeleton led to a decreased cell stiffness when α-DG was stimulated compared to untreated cells with an intact actin cytoskeleton^201^. This suggests that the DGC is involved, to some extent in regulating viscoelasticity of striated muscle tissue, although this needs further clarification, particularly for cardiomyocytes.

### The molecular mechanisms underpinning cardiac dysfunction in the DMD patient

The molecular pathogenesis underpinning DMD-associated DCM can attributed to several key stages: i) structural integrity of the cardiomyocyte sarcolemma is compromised^9^, ii) Ca^2+^ dysregulation caused by influx via sarcolemmal microtears and dysregulated ion channels^202^, iii) Disruption to both the actin and microtubule cytoskeleton, resulting in aberrant mechanotransduction^125^, iv) the generation of X-ROS^53^, and v) mitochondrial dysfunction leading to necrosis of the cell^203^. These dysfunctional molecular pathways contribute to the overall DMD cardiac phenotype in a synergistic manner, each exacerbating the next.

#### The cardiac sarcolemma is compromised in DMD

As well as being a signalling hub, dystrophin’s primary role is to buffer biomechanical forces at the cell-ECM interface and redistribute these within the cell^10^. This was evidenced by Le et al. where it was shown that the central rod domain of dystrophin acts as a molecular spring keeping forces below 25 pN over an 800 nm length^10^. This provided strong evidence that dystrophin buffers against excessive forces to maintain the integrity of the sarcolemma as well as supporting its role in mechanotransmission.

Evidence in support of sarcolemmal fragility and how dystrophin acts as a mechanoprotector of the cardiac sarcolemma derives from studies using dyes that are usually not permeable to intact membranes. For example, stress testing the diaphragm using the *mdx* model revealed increased absorbance of the sarcolemmal impermeable dye, porcion orange, compared to WT, highlighting sarcolemmal fragility^9^. Similarly, ex vivo biomechanical stress in dystrophin-deficient myocardium of *mdx* models showed increased uptake of Evan’s blue dye, which is only permeable to cardiomyocytes in the presence of sarcolemmal damage^204^. Together, the data demonstrate that the myocardium in *mdx* models are less able to withstand sarcomeric-generated contraction-relaxation forces, as is the case for human patients^10,205^. Decreasing the afterload in vivo attenuated the disruption observed in cardiomyocytes, as shown by decreased uptake of Evan’s blue dye, reinforcing the notion that biomechanical stresses are responsible for cellular damage in DMD^204^.

The use of the artificial membrane sealants poloxamers further supports the role that dystrophin has as a mechanoprotector. Myocardial fibrosis was decreased in both canine^206^ and murine^207^ models of DMD when poloxamer 188 (P188) was given, supporting the notion that dystrophin buffers the sarcolemmal against excessive biomechanical forces. Furthermore, there was a decrease in left ventricular remodelling, a decrease in serum cTNI and BNP biomarkers, and no uptake of Evan’s blue dye when P188 was administered^208^. These studies highlight that in the absence of dystrophin the sarcolemma of cardiomyocytes is destabilised and vulnerable to biomechanical stress. However, as Townsend reported, the compliance of isolated cardiomyocytes from their canine model did not improve^206^. This raises questions regarding the true efficacy and mechanism(s) underpinning P188 therapy in humans. P188 is FDA approved for short-term use but clinical trials examining its efficacy in treating progressive DCM and skeletal muscle dysfunction in DMD patients are still underway, but in conjunction with other pharmacological therapies, P188 may be able to attenuate the disease phenotype^209^.

However, in addition to sarcolemmal fragility, there is accumulating evidence in support of dysregulated physiological repair of the sarcolemma in muscular dystrophies^203,210^. Damage to the sarcolemma may be multifactorial in DMD where not only is the sarcolemma structurally weakened, but secondarily to this, sarcolemmal repair mechanisms are dysfunctional, caused by elevated Ca^2+^ influx^211^. In response to sarcolemmal injury, mitochondria have been shown to translocate and bind to micro-tears in the sarcolemma, thereby initiating repair^212^. It is suggested that the localisation of mitochondria to sites of damaged sarcolemma is to function to ‘soak’ up excess Ca^2+^. Indeed, it has been shown in muscular dystrophies, sustained Ca^2+^ overload results causes mitochondrial dysfunction, resulting in poor sarcolemmal injury-repair^212^.

Sustained Ca^2+^ overload promotes a permeability transition in mitochondria causing these to develop a large pore complex that promotes autophagy of mitochondria and cell death^203^. A key component associated with permeability transition in mitochondria is cyclophilin D that causes mitochondrial rupture if not rapidly reversed. The genetic and pharmacological inhibition of cyclophilin D mitigated mitochondria sensitivity to Ca^2+^ overload and prevented swelling^203^. Overall, the authors showed that this was sufficient to prevent mitochondrial driven necrosis.

#### Ca^2+^ is a potent secondary mechanism contributing to the pathogenesis in DMD

Mounting evidence supports the deregulation of calcium homeostasis in the absence of a functional DGC complex^202,213^. Not only is Ca^2+^ pivotal for excitation-contraction coupling of cardiomyocytes but is also a significant player as a secondary signalling ion; therefore, it is no surprise that [Ca^2+^]_i_ is tightly controlled in cardiomyocytes. There is substantial evidence that supports increased Ca^2+^ entry into cardiomyocytes in DMD causing activation of proteases^214^, mitochondrial dysfunction^215,216^, generation of X-ROS^53,217^, promotion of necrosis^192,218^, and aberrant mechanotransduction^167^.

There is some debate as to how Ca^2+^ enters the cell with two main, non-mutually exclusive, propositions: i) Influx of extracellular Ca^2+^ down its concentration gradient into the cardiomyocyte via microtears in the sarcolemma^219^, ii) dysregulation of mechanosensitive Ca^2+^ ion channels (including TRPC, LTCC, and stretch-activated channels) which may be modulated via the microtubule cytoskeleton^15,53^. What is evident, however, is that once Ca^2+^ overload becomes established in cardiomyocytes, it propels the cardiac phenotype observed in DMD^202,220,221^.

Dysregulated Ca^2+^ flux affects sensitive ion channels including ryanodine receptors (RyR2) located at the sarco/endoplasmic reticulum Ca^2+^ ATPase (SERCA) of cardiomyocytes. RyR2 are triggered to release additional Ca^2+^ in response to Ca^2+^ influx, a process termed calcium-induced calcium release (CICR). In *mdx* mice, RyR1 receptors were shown to be hypernitrosylated and prone to increased Ca^2+^sparks^222^. RyR2 of cardiomyocytes are also hypernitrosylated in DMD, linking dysregulated Ca^2+^ to ROS production, further exacerbates its activity^223^. Destabilisation of the RyR2 channel in cardiomyocytes causes it to become ‘leaky’, manifesting as arrhythmias. Fauconnier showed that stabilisation of the RyR2 receptor was sufficient to prevent arrhythmia in vivo^223^. Lastly, P188 can normalise Ca^2+^ influx to the cardiomyocyte, providing convincing evidence that sarcolemmal disruption is a bona fide entry mechanism for Ca^2+^ and that aberrant CICR is a trigger of fatal arrhythmias in DMD^207^.

Mechanosensitive stretch-activated calcium channels, such as the TRPC family of cation channels, are dysfunctional in DMD^220^; being hypersensitive to stress-stimulated contraction (SSC) producing an augmented response to systolic contraction that is critical to the pathogenesis in DMD^112^. TRPC6 was suggested to be mechanosensitive and its activity can be downregulated by protein kinase G (PKG), that in turn admonishes the SSC response^112^. Hyperactive SSC has been linked to arrhythmias in DMD which is underpinned by TRPC6 activation^112^. This study nicely connects mechanosensitive Ca^2+^ dysregulation to the arrhythmias and sudden cardiac death observed in patients with DMD. Moreover, TRPC6 dysregulation is linked to elevated s-nitrosylation in cardiomyocytes, including cysteine residues on SERCA^113^. Deletion of TRPC6 gene in the double knockout murine model *mdx:utrn*^*+/-*^ broadly reversed the cardiac pathology by decreasing hyper-s-nitrosylation, decreasing Ca^2+^, and improving cardiac remodelling^113^.

Pharmacological inhibition of TRPC6 and TRPC3 in DMD vascular smooth muscle cells using GsMTx-4, a mechanosensitive ion channel inhibitor, attenuated the elevated, pathological, [Ca^2+^]_i_^114^. The authors demonstrated a reduction in NADPH oxidase 2 (NOX2) activity with a concomitant decrease in ROS, attributed to GsMTx-4 activity, however, the exact mechanism was not fully described^224^. Elsewhere, it has been shown that GsMTx-4 can be cardioprotective and therefore this may represent a pharmacological mechanosensitive therapeutic strategy for DMD^225^.

#### The cytoskeleton is dysfunctional in DMD and is a key contributor to the mechanopathogenesis of cardiomyocytes

As described previously, the cytoskeleton has a significant role in maintaining the homeostasis of the cardiomyocyte, with mechanotransduction playing centre stage in many cellular processes^1,195,226^. Therefore, disruption of the cytoskeleton is likely to significantly impact the overall functionality of the cell, and indeed this is the case in DMD.

In particular, the microtubule cytoskeleton has generated widespread interest in the context of DMD^53,125,227^. Dystrophin directly interacts with microtubules specifically at the spectrin repeat 24 and the WW domain^125^. The absence of dystrophin disrupts the microtubule lattice, with *mdx* mice demonstrating a 2.5-fold increase in α-, and β-tubulin monomers, suggesting disorganisation of microtubules^125^. Interestingly, elevated tubulin monomers in *mdx* did not correlate to a shift in the balance of tubulin-microtubule equilibrium, but rather the long-term stabilisation of microtubules was disrupted^125^.

In DMD, the microtubule cytoskeleton was reportedly stiffer compared to WT controls^53^, immediately suggesting alterations to the mechanobiology, especially considering the tensegrity model. Functionally, it has been reported that mechanical stretch increases NOX2 generated X-ROS as well as elevated Ca^2+^ in *mdx* but not in WT muscles^228^. The role of the microtubule network, in connecting axial stress, with NOX2, and Ca^2+^ is particularly significant as it relates all of the core pathological features in DMD. The authors suggested that either Piezo 1/2 or TRPC1 stretch activated channels were responsible for the influx of Ca^2+^ observed, that as previously described is a significant contributor to DMD pathogenesis^228^.

Posttranslational modification of the cytoskeleton has revealed itself to be an additional mechanism underpinning DMD^53^. De-tyrosination of α-tubulin was central in disrupting the microtubule cytoskeleton in DMD^53^ increased the stiffness of microtubules, disrupting the ability of cells to mechanosense and respond to their environment^53^. Ultimately, disruption to microtubules was a prominent driver in *mdx* cardiac related death^53^. Parthenolide, which decreases detyrosination of α-tubulin, can significantly improve the cardiac phenotype in *mdx*, where 100% of treated mice survived an isoproterenol challenge compared to <10% of untreated *mdx* mice^53^. Moreover, parthenolide prevented aberrant Ca^2+^ waves in response to stress, underscoring the interaction between the cytoskeleton to Ca^2+^ regulation^53^. Overall, these findings implicate the disorganisation of the cytoskeleton as being critical determinant of DMD pathogenesis.

Microtubule costameric disorganisation in DMD is also related to organelle mislocalisation, for example, the Golgi complex^229^. The Golgi apparatus was shown to be mislocalised with distinct morphological characteristics in *mdx* compared to WT, features that also correlated to aberrant posttranslational modifications of proteins^229^. The authors successfully rescued the disease phenotype by transfecting *mdx* skeletal muscle cells with the micro-dystrophin, ΔR4-R23, containing binding motifs to actin and microtubules^229^.

Furthermore, the localisation of the nucleus may also be affected as reported by Iyer, with its movement being significantly elevated in *mdx* compared to WT mice^227^. Interestingly, Iyer showed significant disruption to the LINC complex of *mdx* mice with the majority of the central LINC complex proteins (nesprin, SUN1/2, emerin, lamin A/C) being downregulated^227^, thereby reducing the connection between the nucleus and cytoskeleton, a feature in itself can be a cause for muscular dystrophies and aberrant mechanotransduction^230–232^. They also observed decreased transcriptional activity in LINC complex proteins, notably in the gene for nesprin 1, *Syne 1*^227^. Together these findings suggest that disruption to the microtubule cytoskeleton is a significant contributing factor towards the mechanopathogenesis in DMD. The majority of the findings have been reported for skeletal muscle tissue, and further clarification of the role microtubules have in cardiomyocytes is of paramount importance.

The actin cytoskeleton is also central to the mechanobiology of cardiomyocytes, being largely responsible for cell stiffness and propagation of force as mechanical waves^120^. Reports in hiPSC-derived cardiomyocytes showed that restoration of the ABD1/2 binding sites of dystrophin significantly improved Ca^2+^ handling dynamics^233^ suggesting that the interaction between dystrophin and actin is important in regulating calcium dynamics. In support of this notion, mutations in cytoskeletal genes, including those of the DGC (*DMD, PDLIM3, FKTN, SGCG*, and *SSPN)* are a cause for atrial fibrillation in inherited DCM^234,235^. Fatal tachyarrhythmias are pathognomonic in DMD, and it is interesting that mutations of the cytoskeleton seem to be so strongly associated with this phenotype.

The γ-actin subsarcolemmal lattice directly interacts with dystrophin and was found to be increased 10-fold in *mdx* mice compared to WT controls^236^, potentially a compensatory mechanism in attempt to maintain the integrity of the subsarcolemmal lattice, however the connection to the F-actin deeper within the cell is disrupted, thereby negatively impacting mechanotransduction along prestressed actin cables. Upregulation of γ-actin complements the increased, compensatory, expression of α7β1 described previously in DMD patients^147^.

Lastly, it has been shown that changes in the epigenetic regulation of the actin cytoskeleton and cardiac remodelling are another key component of DMD^237^. Elevated expression of the nucleoporin (NUP) 153 was increased in *mdx* model and found to be acetylated, activating its function, and driving gene transcription in cardiomyocytes promoting cardiac remodelling^237^. Actin-binding protein genes, including nexilin, were increased by NUP 153 as well as the expression and function of Ca_V_1.2 ion channels, promoting arrhythmias^237^. Increased expression of NUP 153 was validated by Nanni in human DMD cardiac samples, implicating a role for disrupted epigenetic regulation of the cytoskeleton^237^.

## Conclusions

The importance of the DGC in the maintenance of striated muscle tissue cannot be understated. In its absence, patients suffer from a catastrophic, life-limiting muscular dystrophy, impacting all aspects of their lives. In order to alleviate the disease phenotype, with the aim of curing patients with DMD it is important to understand and examine the underpinning mechanisms.

The myocardium must have mechanisms to protect against the sarcomeric generated forces to prevent contraction-induced injury, especially considering that it contracts from birth through to death. Dystrophin is a principal agent in the protection against contraction-induced forces, facilitating sarcolemmal integrity, as well as being a scaffold for mechanosensitive proteins. Absence of dystrophin and/or the DGC renders the sarcolemma of cardiomyocytes incredibly fragile and unable to withstand contraction-induced injury, resulting in the pathogenesis observed in DMD. Moreover, it has been revealed that the underlying sarcolemmal repair mechanisms are themselves disrupted, further exacerbating the integrity of the cell.

Disruption to the vital connection between cytoskeleton and ECM at costameric regions, causes disruption to mechanotransduction as well as mechanical dysfunction at the cellular level. These serve to promote increased susceptible to mechanical stimuli, in turn, leading to increased dilatation and progressive DCM of the heart.

Examination into the central proteins of the DGC alongside their interaction to the cytoskeleton, mechanosensitive proteins (e.g. YAP), the ECM, the nucleus, is paramount to understanding the disease. How these changes translate into disruptions in the mechanical signalling pathways, gene expression, and overall organ function still remains to be fully explored.

Examination into biomechanics has revealed itself to be particularly important in governing cellular and molecular processes, that go onto dictate higher order phenotypes. In particular, we have highlighted and examined evidence that showcases the impact of disrupted biomechanics in DMD and how it is a central driver for the disease pathogenesis. Overall, the absence of dystrophin, and indeed other constituents of the DGC, weakens the sarcolemma, rendering it susceptible to contraction-induced injury with a negative impact on cellular mechanotransduction, mitochondrial dysfunction, pro-inflammatory and necrotic cell death being characteristic. Moreover, faulty ion channel regulation, elevated Ca^2+^, and ROS production, further drive the pathology and can account for arrhythmias and sudden cardiac death observed in patients. Long-term changes are communicated to the nucleus and aberrant mechanotransduction promotes altered gene expression.

In summary, it is crucial to consider the important role that biomechanics has in regulating cellular and molecular physiology and how it can be a leading contributor to disease progression. Here, we have demonstrated the importance of biomechanics in DMD, however, much work is still required to define and tease apart the mechanisms more clearly, particularly in integrating the different topics discussed to achieve beneficial therapeutic and life changing outcomes for patients.

### Reporting summary

Further information on research design is available in the Nature Research Reporting Summary linked to this article.

## Supplementary information

reporting summary
